# Appropriateness of reference genes for normalizing messenger RNA in mouse 2,4-dinitrobenzene sulfonic acid (DNBS)-induced colitis using quantitative real time PCR

**DOI:** 10.1038/srep42427

**Published:** 2017-02-10

**Authors:** Nour Eissa, Laëtitia Kermarrec, Hayam Hussein, Charles N. Bernstein, Jean-Eric Ghia

**Affiliations:** 1 Immunology, University of Manitoba, Winnipeg, MB, Canada; 2 Children’s Hospital Research Institute of Manitoba, University of Manitoba, Winnipeg, MB, Canada; 3 Large Animal Medicine, William R. Pritchard Veterinary Medical Teaching Hospital, School of Veterinary Medicine, University of California Davis, CA, USA; 4 Internal Medicine section of Gastroenterology, University of Manitoba, Winnipeg, MB, Canada; 5 IBD Clinical and Research Centre, University of Manitoba, Winnipeg, MB, Canada

## Abstract

2,4-Dinitrobenzene sulfonic acid (DNBS)-induced colitis is an experimental model that mimics Crohn’s disease. Appropriateness of reference genes is crucial for RT-qPCR. This is the first study to determine the stability of reference gene expression (RGE) in mice treated with DNBS. DNBS experimental Colitis was induced in male C57BL/6 mice. RNA was extracted from colon tissue and comprehensive analysis of 13 RGE was performed according to predefined criteria. Relative colonic *TNF-α* and *IL-1β* mRNA levels were calculated. Colitis significantly altered the stability of mucosal RGE. Commonly used glyceraldehyde-3-phosphate dehydrogenase (*Gapdh*), β-actin (*Actb*), or β2-microglobulin (*β2m*) showed the highest fluctuation within the inflamed and control groups. Conversely, ribosomal protein large P0 (*Rplp0*), non-POU domain containing (Nono), TATA-box-binding protein (*Tbp*) and eukaryotic translation elongation factor 2 (*Eef2*) were not affected by inflammation and were the most stable genes. *TNF-α* and *IL-1β* mRNA levels was dependent on the reference gene used and varied from significant when the most stable genes were used to non-significant when the least stable genes were used. The appropriate choice of RGE is critical to guarantee satisfactory normalization of RT-qPCR data when using DNBS-Model. We recommend using *Rplp0, Nono, Tbp, Hprt* and *Eef2* instead of common reference genes.

Inflammatory bowel diseases (IBDs) are idiopathic, chronic, and relapsing gastro-intestinal inflammatory disorders that are characterized by abdominal pain, weight loss and diarrhea. Ulcerative colitis (UC) and Crohn’s disease (CD) are the two main forms of IBD and they have both overlapping and distinct clinical and pathological features; however, they can be distinguished by the location of the inflammation within the gastro-intestinal (GI) tract^1^. The etiology of IBD is unknown, but there are several factors that may contribute to its pathophysiology such as genetic factors, immune system dysregulation, microbial dysbiosis, stress, and disruption of tight junctions. To investigate these different etiological factors, many experimental models are available and include chemical-induced and transgenic animal models, spontaneous models and genetically engineered^2^. Animal models of IBD are a main source of information about the pathophysiology and are clinically relevant to both human UC and CD.

Because of their low cost and rapid onset of disease, chemical-induced colitis models are considered valuable tools to study various aspects of IBD. The dextran sulfate sodium (DSS) model, a model of injury-repair mimicking UC, is one of the most common chemical models used to induce colitis in rodents^3^, but the model needs to be well-controlled to avoid variation in DSS concentration, and inconsistent water uptake by mice resulting in uneven exposure to DSS, in which causes variation in the degree, extent, and distribution of mucosal damage in the colon^4^. Several models can mimic CD^5^, but the two main models are the hapten-induced trinitrobenzene sulfonic acid (TNBS) and the dinitrobenzene sulfonic acid (DNBS) models of colitis, where the agents are given *via* rectal instillation, diluted in varying concentrations of ethanol^6^. Ethanol administration is required to disrupt the colonic mucosal barrier to allow penetration of DNBS or TNBS into the lamina propria and to haptenize the localized colonic and gut microbial proteins to become immunogenic, and activate the host immune responses^7^. DNBS can bind covalently to the E-amino group of lysine and alter cell surface proteins to be haptenated proteins, which induce the release of interleukin-12 (IL-12), the activation of the T-helper 1-mediated local immunological responses and the activation of macrophages to overproduce proinflammatory cytokines such as interferon-gamma (INF-γ), interleukin (IL)-β, IL-12, tumor necrosis factor (TNF)-α and nitric oxide (NO) due to induction of inducible nitric oxide synthase (iNOS). These activations contribute to the inflammatory process that in turn result in transmural inflammation reflected by weight loss and diarrhea^6^,^8^,^9^. Clinically, DNBS causes severe inflammation in the colon and rectum^10^, and induces a strong inflammatory response that is associated with a significant increase in myeloperoxidase (MPO) activity and overproduction of IL-1β and TNF-α^11^. Compared to DNBS, TNBS is considered to be a hazardous chemical because of its highly oxidative properties that can increase the risk of explosion upon contact with bases such as sodium and potassium hydroxide. Moreover, DNBS binds more selectively to proteins than TNBS, binding only to the ε-amino group of lysine^6^. Furthermore, under its powder form, TNBS is no longer accessible in the United States of America^6^. Consequently, DNBS is currently favored over TNBS to induce colitis.

Quantitative real-time polymerase chain reaction (RT-qPCR) is a powerful technique used to increase our understanding of the molecular pathophysiology of IBD, and it is characterized by a high sensitivity, a relatively low cost, and an high time efficiency^12^. RT-qPCR accuracy is affected by the stability of the reference genes. RT-qPCR data analysis includes reference genes in the comparative cycle threshold (Ct) method^13^. The optimal reference gene should be characterized by stable messenger RNA (mRNA) expression in all samples studied, regardless the tissue type, the disease state, or the experimental conditions, and it should have expression levels comparable to that of the target gene^14^. Therefore, careful selection, evaluation and validation of the optimal reference gene are vital for achieving reliable results and to avoid possible data inaccuracies from the use of a suboptimal reference gene.

Recently, we reported the stability of reference genes in mouse DSS- experimental colitis^15^, which is the most common and widely used injury/repair animal model of colitis. Several studies used DSS and DNBS animal models to address their research questions^16^,^17^,^18^,^19^,^20^,^21^,^22^. Therefore, in the current study, we performed the first comprehensive study of reference gene stability in a DNBS model of colitis to differentiate between optimal and suboptimal reference genes and to investigate the problems associated with using non-validated or unsuitable reference genes and their impact on data accuracy. Evaluation of 13 potential reference genes (*Gapdh, Actb, β2m, Hmbs, Hprt, RpLp0, Tbp, Gusb, Ppia, Oaz1, Nono, Tfrc*, and *Eef2*) was assessed in colitic mice by analyzing reference gene stability using different algorithms such as geNorm^23^, bestKeeper^24^, normFinder^25^, the comparative delta Ct method^26^, and the comprehensive ranking^27^. These approaches provided a detailed comprehensive analysis of common and novel reference genes for the use of mouse DNBS-induced colitis in relation to RT-qPCR experiments. Moreover, to validate the influence of reference gene stability on target gene normalization, the relative gene expression of two major colonic pro-inflammatory cytokines *TNF-α* and *IL-1β* in inflamed and non-inflamed colonic tissues was evaluated using the 13 housekeeping genes to find the most suitable endogenous normalizer genes for mRNA expression.

## Results

### Confirmation of DNBS – Induced Colitis

**Figure 1:**
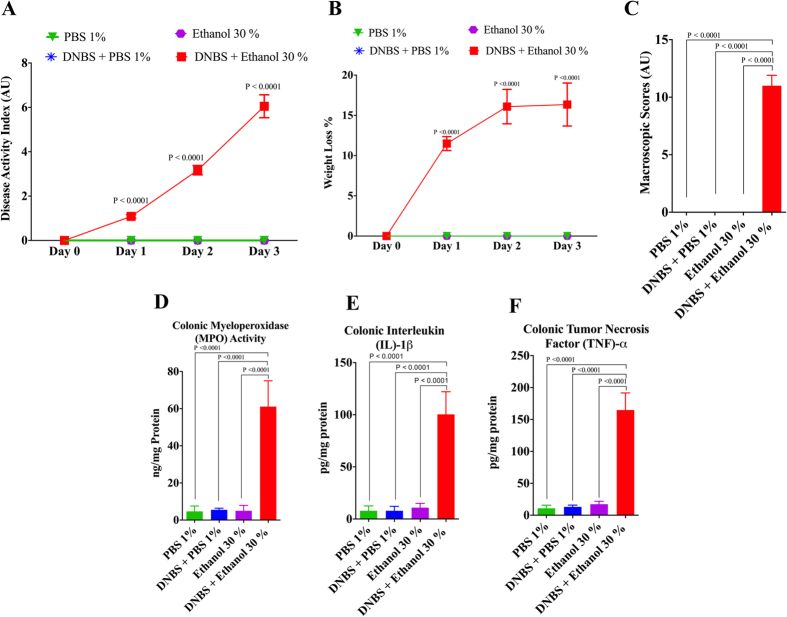
Confirmation of colitis induction by dinitrobenzene sulfonic acid (DNBS). (**A**) Disease activity index and (**B**) weight loss percentage were analyzed by repeated measures ANOVA analysis followed by the multiple comparisons post hoc analysis; (**C**) macroscopic scores. (**D**) Myeloperoxidase (MPO) activity in the colon; and colonic pro-inflammatory mediators: (**E**) IL-1β, (**F**) TNF-α. The control groups (PBS 1%, DNBS + PBS 1%, Ethanol 30%), while DNBS-induced colitis group (DNBS + Ethanol 30%), n = 6/group. One Way ANOVA analysis followed by the multiple comparisons post hoc analysis were used to compare the experimental groups. Data are presented as the mean ± SD.

Induction and development of experimental colitis was confirmed in the DNBS-treated group compared to controls. Based on the DAI, the weight loss and the macroscopic scores (Fig. 1A,B and C), the DNBS-treated group (DNBS + Ethanol 30%) showed a significant increase of inflammation compared to the control groups (PBS 1%, DNBS + PBS 1%, Ethanol 30%). This was confirmed by a 15-fold increase in the protein level of colonic MPO activity (Fig. 1D). The protein levels of colonic pro-inflammatory cytokines (IL-1β, TNF-α) were also significantly increased by 10 - fold and 17 - fold change respectively (Fig. 1E,F).

### Primer specificity and efficiency

The performance for each primer set *via* primer specificity and efficiency was assessed. The dissociation curve following RT-qPCR confirmed the amplicon specificity. A single peak in the melting curve analyses for each of the 15 sets of primers indicated high specificity. The amplification efficiency for all selected primers ranged from 91% to 125% (Table 1) and the correlation coefficients (R^2^) were equal or greater than 0.99 (Fig. S1).

### Reference gene expression profiles

The expression levels of the 13 reference genes were evaluated using Ct and descriptive statistics (mean, SD, median, min, max) (Table 2) in all tested samples. The mean Ct values for reference genes ranged between 14 and 38, with most between 18 and 25. *Oaz1* had the highest median Ct value (38.14), which indicated a relatively low expression at the colon level, while *Actb* had the lowest median Ct value (Ct = 14.74), which indicated relatively high expression at the colonic tissue. The lowest standard deviation was determined for *Tbp* (SD = 0.7309) *Rplp0* (SD = 0.7325), *Hprt* (SD = 0.9832), *Eef2* (SD = 1.405) and *Nono* (SD = 1.431), defining these two reference genes as having the lowest variability, while *Gapdh* (SD = 1.511), *Actb* (SD = 1.634), *Hmbs* (SD = 1.637), *Ppia* (SD = 1.761), *Gusb* (SD = 1.808), *Oaz1* (SD = 1.808), *β2m* (SD = 1.897) and *Trfc* (SD = 2.095) were defined as having the highest instabilities in their mRNA expression.

### Effect of inflammation on selected reference gene mRNA expression in colonic mucosa

**Figure 2:**
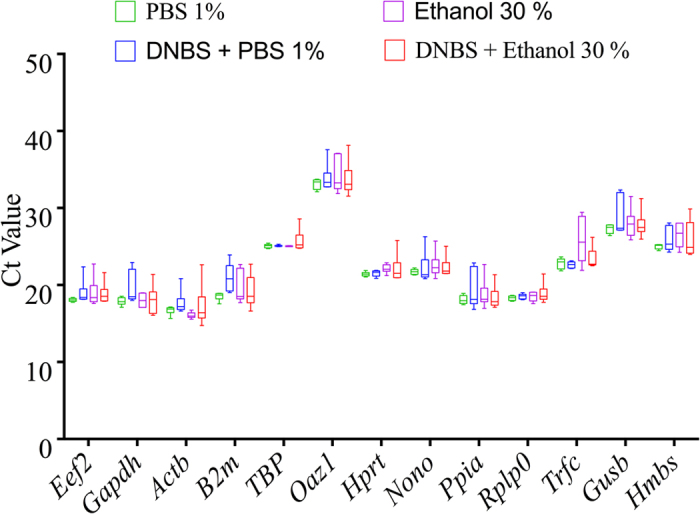
Effect of inflammation on candidate reference gene expression in colonic mucosa. DNBS-induced colitic (DNBS + Ethanol 30%) and non-inflamed control colon groups (PBS 1%, DNBS + PBS 1%, Ethanol 30%) are defined in the Materials and Methods (n = 6/group). One Way ANOVA analysis followed by the multiple comparisons post hoc analysis were used for comparison between the groups. Ct, threshold cycle.

To evaluate whether inflammation had any effect on mRNA expression levels, the candidate gene mRNA from the DNBS group compared to the non-colitic control group were determined. As described in Fig. 2, threshold cycle (Ct) values of all reference genes were not significantly altered under inflammatory conditions. Although there is no significant alteration between colitic (DNBS + Ethanol 30%) and non-colitic groups (PBS 1%, DNBS + PBS 1%, Ethanol 30%) for most of reference genes, a high variability was shown by the high standard deviations for *Actb, β2m, Trfc, Gapdh and Hmbs*.

### Appropriateness of reference genes

To examine and rank reference gene appropriateness, five different tools were used to calculate the expression stability of selected reference genes: NormFinder, Comparative Delta CT, geNorm gene, BestKeeper gene stability, and final comprehensive gene stability ranking.

#### Normfinder gene stability

**Figure 3:**
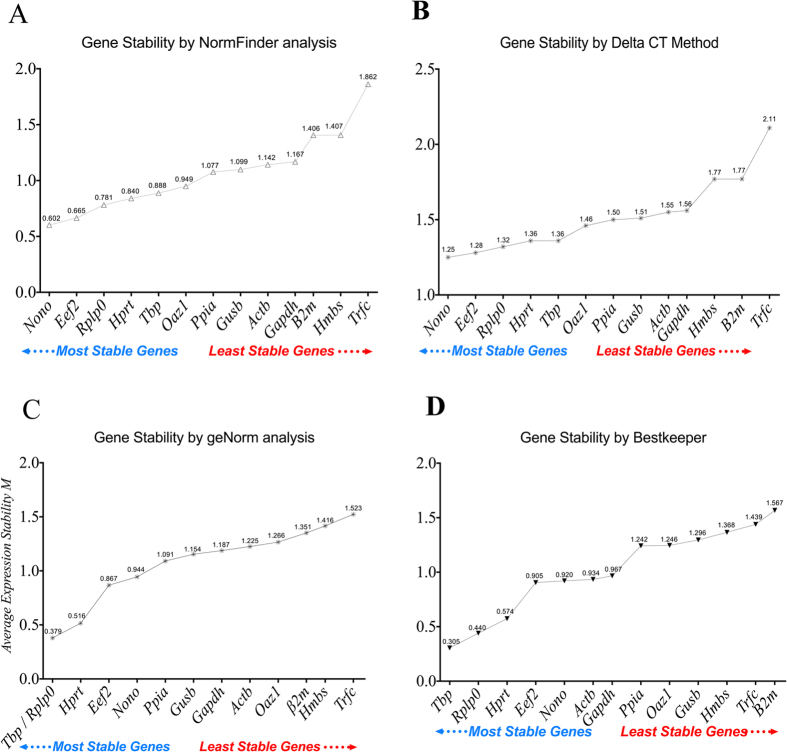
Gene expression stability values of the 13 reference genes analyzed by defined criteria (**A**) NormFinder (**B**) Delta CT Method (**C**) geNorm Analysis (**D**) BestKeeper analysis for 24 colon samples from the control groups (PBS 1%, DNBS + PBS 1%, Ethanol 30%), while DNBS-induced colitis group (DNBS + Ethanol 30%), n = 6/group. Lower values refer to higher stability and higher values refer to a lower stability.

Figure 3A shows the ranking order of the 13 candidate reference genes mentioned above, using the NormFinder program to calculate their expression stability. Genes that are more stably and optimally expressed are indicated by lower average expression stability values. The analysis ranks the selected candidate from most stable gene to least stable; *Nono, Eef2, Rplp0, Hprt and TbP* were most stable genes while, *Trfc, Hmbs, β2m, Gapdh and Actb* were least stable genes.

#### Comparative Delta CT

In the present study, the average expression stability of all selected reference genes was calculated by comparative delta CT (Fig. 3B). Classical reference genes such as *Trfc* (2.11), *Hmbs* (1.770), *β2m (1*.*770*), *Gapdh* (1.560) *and Actb* (1.550) had the highest expression instability while *Nono* (1.25), *Eef2* (1.28), *Rplp0* (1.32), *Hprt* (1.36), and *Tbp* (1.36) were most stable genes in the colonic tissue.

#### geNorm analysis

Average expression stability (M value) of all genes was calculated by geNorm software. The M values of the candidate reference genes across all samples are shown in Fig. 3C. Lower values refer to high stability, geNorm ranked the selected genes from most to least stable gene, as follow *Tbp*/*Rplp0* (0.379), *Hprt* (0.516), *Eef2* (0.867), *Nono* (0.944), *Ppia* (1.091), *Gusb* (1.154), *Gapdh* (1.187), *Actb* (1.225), *Oaz1* (1,266), *β2m* (1.351), *Hmbs* (1.416) and *Trfc* (1.523). The two members set *TbP*/*Rplp0* are excellent optimal reference genes that were selected by geNorm.

#### BestKeeper Analysis

The reference gene evaluation by BestKeeper tool is shown in Fig. 3D. The best keeper revealed that in DNBS induced colitis the highest stable genes were *Tbp* (0.305), *Rplp0* (0.440), *Hprt* (0.574), *Eef2* (0.905) and *Nono* (0.920), and while the least stable genes were *Actb* (0.934), *Gapdh* (0.967), *Ppia* (1.242), *Oaz1* (1.246), *Gusb* (1.296), *Hmbs* (1.368), *Trfc* (1.439) and *β2m* (1.567).

#### Comprehensive gene ranking

**Figure 4:**
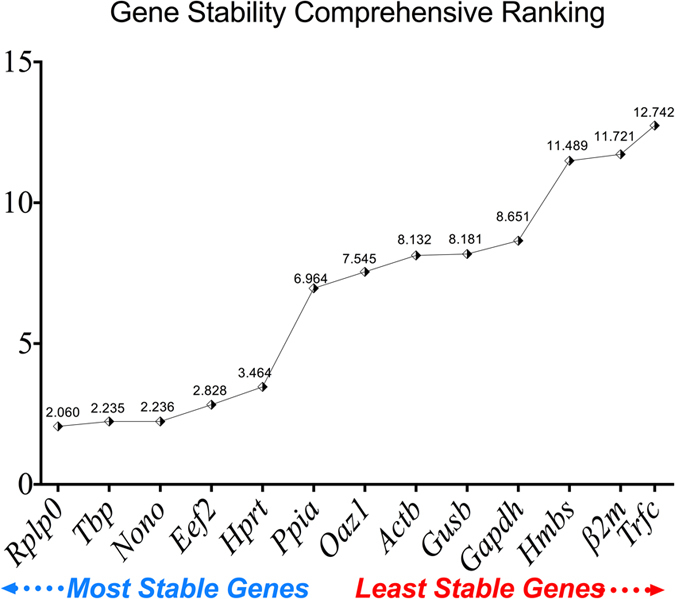
Comprehensive gene stability ranking for the 13 reference genes used in DSS-induced colitis and control groups. RT-qPCR was carried out for each of the 13 reference genes using the same RNA samples from control groups (PBS 1%, DNBS + PBS 1%, Ethanol 30%) and DNBS-induced colitis group (DNBS + Ethanol 30%). CT values were used to calculate the comprehensive gene stability ranking.

Taking in consideration all the previous gene-ranking tools NormFinder, Comparative delta CT, geNorm and BestKeeper analyses, reference gene stability was calculated using the overall comprehensive ranking system. As shown in Fig. 4, comprehensive gene ranking from the most stable to the least stable gene is as follows: *Rplp0* (2.060), *TbP* (2.235), *Nono* (2.236), *Eef2* (2.828), *Hprt* (3.464), *Ppia* (6.964), *Oaz1* (7.545), *Actb* (8.132), *Gusb* (8.181), *Gapdh* (8.651), *Hmbs* (11.489), *β2m* (11.721) and *Trfc* (12.742). Data from the tools used showed that *Gapdh, Actb*, and *β2m* are suboptimal candidates for normalization of target gene expression in the DNBS-model because their stability was affected by the presence of inflammation and the experimental conditions. However, *Nono, Rplp0, TbP*, and *Eef2* showed consistent expression stability.

### Influence of reference gene choice on the target mRNA relative expression

**Figure 5:**
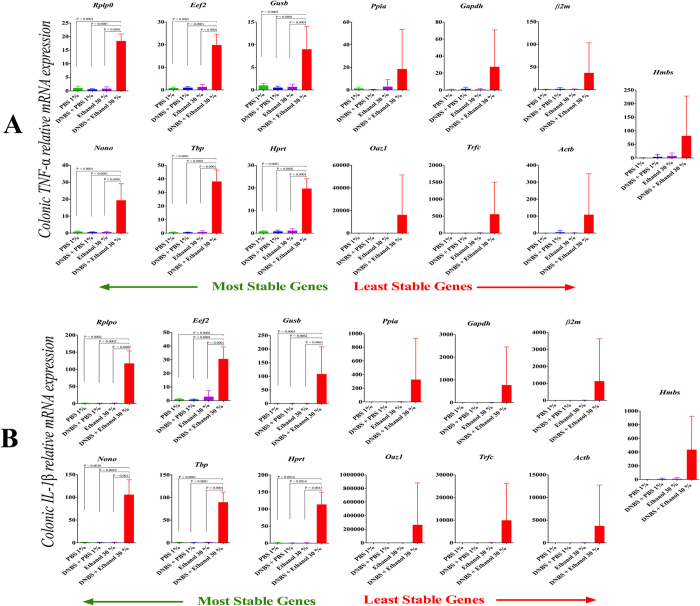
Effect of reference gene selection on the relative expression of colonic *TNF-α* (**A**) and *IL-1β* (**B**). Target gene expression was calculated against the 13 reference genes using comparative the ΔΔCt method. Significant changes between control groups (PBS 1%, DNBS + PBS 1%, Ethanol 30%) and DNBS-induced colitis group (DNBS + Ethanol 30%). were seen only with the stable reference genes. One Way ANOVA analysis followed by the multiple comparisons post hoc analysis were used for comparison between the groups. Data is presented as the mean ± SD, (n = 6/group).

To determine if the choice of reference gene used to normalize the gene of interest expression significantly alters the statistical outcome reported, normalization of *TNF-α* and *IL-1β* gene expression in DNBS model was investigated using comparative the ΔΔCt method (Fig. 5). The present study shows that reference gene selection significantly affects mRNA expression levels of *TNF-α* and *IL-1β*, which can substantially alter the results and their associated interpretation. The magnitude of the relative fold change and the standard error of the mean were amplified when the genes of interest were normalized against the least stable gene candidate, as we expected. When normalized against the sub-optimal or least stable genes, colonic and *IL-1β* expression levels (Fig. 5A,B) had a higher variability in DNBS-experimental colitis (DNBS + Ethanol 30%), which shifted the results from a significant up-regulation to a non-significant up-regulation. The P value changed with different reference genes when inflamed (DNBS + Ethanol 30%) and control groups were compared (PBS 1%, DNBS + PBS 1%, Ethanol 30%). Using the least stable genes, no significant difference in *TNF-α* and *IL-1β* mRNA levels was reported (*Hmbs, Actb, Gapdh, Trfc, Ppia, Oaz1, β2m*). However, the relative mRNA expression levels were significantly up regulated in the inflamed colon when reference genes associated with a high stability were used (*Rplp0, Hprt, Eef2, Tbp, Nono, Gusb*).

### Reference gene stability in DNBS-induced experimental colitis using the External RNA Controls Consortium (ERCC)

**Figure 6:**
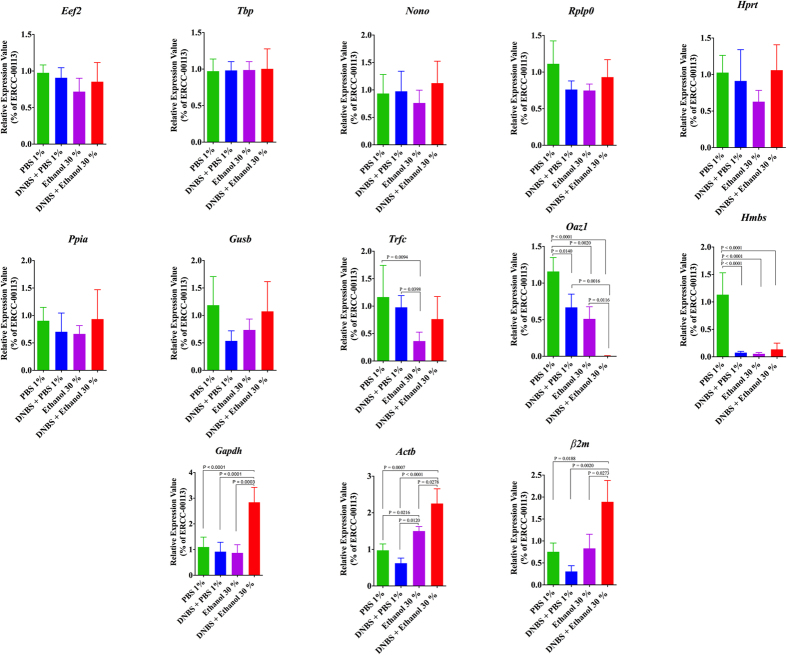
Alterations in the expression levels of the potential reference genes in colon of control groups (PBS 1%, DNBS + PBS 1%, Ethanol 30%) and DNBS-induced colitis group (DNBS + Ethanol 30%). The expression levels of these genes in colonic samples were normalized to the Ct values of ERCC-00113, an external control RNA. The graphs show relative mRNA expression values that calculated using the ΔΔCt method. One Way ANOVA analysis followed by the multiple comparisons post hoc analysis were used to compare the groups with significance level 0.05. Data are shown as mean ± S.D. (n = 6/group).

To gain more confirmatory insights, the mRNA levels of the 13 selected reference genes in control and DNBS-groups were calculated based on the Ct values of control ERCC RNA (*ERCC-00113*). The expression levels of the least stable genes (*Actb, Gapdh, Hmbs, Oaz1, β2m, Trfc*) in the DNBS experimental colitis group (DNBS + Ethanol 30%) were significantly down regulated or unregulated compared to the control groups (PBS 1%, DNBS + PBS 1%, Ethanol 30%) while the most stable genes (*Rplp0, Nono, Eef2, Tbp, Hprt, Ppia, Gusb*) did not demonstrate any significant change (Fig. 6). Taken together, these data indicated that the expression of the most stable genes in colonic inflammation induced through DNBS dissolved in 30% ethanol administration is relatively stable compared to the least stable reference genes.

## Discussion

Appropriate reference genes are essential for optimal data normalization and thus generating reliable results in studies of gene expression. Our study is the first systematic report on the appropriateness of reference genes that could be used for RT-qPCR data normalization in the DNBS mouse model. RT-qPCR, with its ability to quantify small amounts of nucleic acids in a wide range of samples from numerous sources, has been used extensively in molecular biology. Normalization is a prerequisite to reduce the tube-to-tube variations caused by variable RNA quality or reverse transcription efficiency. Since endogenous reference genes are universally used to normalize the expression levels of the target genes, determining optimal and stable reference gene is a crucial step in gene expression experiments.

Commonly used reference genes such as *Gapdh, Actb*, and *β2m* are expressed constitutively, are involved in basic housekeeping functions required for cell maintenance, and are commonly selected as reference genes to normalize gene expression studies^15^,^28^,^29^. Recently, several reports have demonstrated that expression of these genes could be altered in different tissues during growth and differentiation, in response to different stimuli, and under pathological conditions^15^,^30^. Therefore, research on alternative genes is required. NormFinder, geNorm, BestKeeper, the comparative ΔCt method, and the comprehensive ranking are popular algorithms to determine the stability of reference genes from a set of selected candidate reference genes under certain experimental conditions.

DNBS-induced colitis is an experimental mouse model that is used to study some features of the pathogenesis of CD, the effects of potential of preclinical therapies, and the mechanisms underlying intestinal inflammation and mucosal injury in the preclinical state^6^,^31^,^32^. Currently, DNBS is favored over TNBS to induce colitis. Currently the use of TNBS is surrounded by many safety issues and concerns raised in the last few years. DNBS is less hazardous than TNBS and can be used safely in a well-ventilated room with personnel wearing protective clothing, gloves and goggles^6^. In our study, we evaluated the suitability of 13 genes, including the most commonly-used reference genes *Gapdh, Actb, Rplp0, β2m*, and *Hprt*, which have been used in normalizing mRNA expression in normal and pathological intestinal mucosa^33^,^34^,^35^.

The thirteen reference genes were selected from Gene bank to analyze their stability by applying different approaches. The commonly used reference genes such as *Gapdh, Actb*, and *β2m* demonstrated significantly high variability, conversely, the most stable genes with the least variability such as *TbP, Rplp0, Nono, Eef2*, and *Hprt* were not affected by the experimental conditions. *TbP* and *Eef2* were shown to be the reference genes of choice for RT-qPCR data normalization when assessing colonic inflammation using the DSS-experimental colitis model^15^. Moreover, *Eef2* is recognized as a constantly expressed reference gene in various mouse tissues and its expression does not significantly vary, nor does it affected by experimental conditions^36^. In the mouse intestine, Tbp has been demonstrated to be an optimal reference gene to normalize gene expression^37^. Also, Rplp0 is the most stable gene in gene expression studies involving human blood CD4 + T cells^38^.

Our results do not recommend the use of the commonly used reference genes such as Gapdh, *β2m*, and Actb. The defect in stability of the classic reference genes can be attributed to their biological functions, which could altered under experimental or disease conditions and expression variations within different tissues. Recent studies demonstrated that *Gapdh, Actb*, and *β2m* did not perform optimally as endogenous control genes in physiological and pathological conditions^15^,^39^. Given the popularity of the DNBS model to study CD, our study demonstrates an important finding and proposes that any new studies researching alternative involvements should include similar validation of a candidate reference gene profile.

To investigate the importance and the impact of normalization using different reference genes on the expression level of target genes, the relative expression of *TNF-α* and *IL-1β* was normalized against 13 reference genes. Overall, our study shows that target gene mRNA levels may be calculated depending on the reference gene selected for normalization, and this could shift the results from significant to non-significant and vice-versa, which ultimately will influence the final interpretation and conclusions, and cause potential inaccuracies in future research. The relative gene expression of *TNF-α* and *IL-1β* followed a similar pattern when either the most stable or the least stable genes were used to normalize gene expression. Comparing inflamed and non-inflamed groups, no statistically significant increase in *TNF-α* and *IL-1β* expression was observed when normalized to the least stable genes. In contrast, *TNF-α* and *IL-1β* gene expression demonstrated a significant increase when normalized to most stable genes. In the current study, we used the ΔΔCt method, and Ct values were normalized to both the reference gene and a control group (in this case the no intervention control group). The use of the least stable reference gene resulted in large gene expression variation and consequently less statistically significant results, highlighting the effectiveness of using a robust analysis of gene stability before beginning any qRT-PCR studies. These findings are supported by previous published studies, which indicate that normalization of a target gene using a sub-optimal or unstable reference gene induced fluctuations in the relative transcript levels of the target gene and caused the final output to be non-significant with high variability in IBD patients^39^, murine corneal model^40^ and circadian studies^41^.

External RNA standards can be used as quality controls for inter-run and cross-platform standardization to strength the correctness of our study forecast based on the panel of biomarkers^42^. In the current study, using the ERCC as an external normalizer to investigate the expression levels of potential reference genes, we demonstrated that inflammatory conditions did not change the expression levels of the most stable reference genes (*Rplp0, Nono, Tbp, Eef2, Hprt*), but they significantly altered that of the suboptimal reference genes such *Gapdh, Actb, Hmbs, Trfc, Oaz1 and β2m*. This may be because mRNA levels of suboptimal reference genes can be changed by metabolic functions under both physiological and pathological conditions. Therefore, in addition to different approaches that can be used to evaluate the appropriateness of reference genes in gene expression experiments, normalization to ERCC RNA standard may be useful as an additional assenting step for clarifying the stability of reference genes.

Many animal models have been developed to characterize the complexity of IBD pathophysiology, to study molecular underlying mechanisms and to determine the potential human therapeutics^43^. Although, DSS is the most popular and widely used mouse model of colonic inflammation^6^, a debate exists regarding the nature of the injury as some experts have classified this model as an injury/repair model and not as UC-model. Overall, this model is easy to use due the route of administration, the simplicity of controlling the dosage, the interval and its cost^44^, however, this model has some limitations related to the variation in DSS concentration, and the inconsistently water uptake results in uneven exposure to DSS, inducing variation in the degree of inflammation^4^. Currently, researchers test their hypothesis using the two different cost effective models as demonstrated in several published articles. Validation of reference genes is critical in RT-qPCR experiments, previously we validated the stability of reference genes in DSS-experimental model^15^. Our previous findings by using DSS model^15^ are supporting the current results using the DNBS experimental colitis. Both models showed least stability of common widely used reference genes (Gapdh, Actb, *β2m*) and most stability with *Tbp, Eef2, Rplp0 and Nono*.

We acknowledge the limitations of the present study. Our findings are valid in, and refer to, colonic tissues isolated from C57BL/6 mice. This study tested the performance of 13 potential reference genes including the most commonly used reference genes, but more optimal reference gene combinations may be defined in the future cannot be omitted. Our findings only apply to the DNBS-model in mice, and, therefore, do not eliminate the use of common reference genes from being optimal reference genes in other experimental conditions, tissues, or species. Consequently, with the development of new models, and application of interventions, validation studies will need to be repeated.

## Conclusion

**Figure 7:**
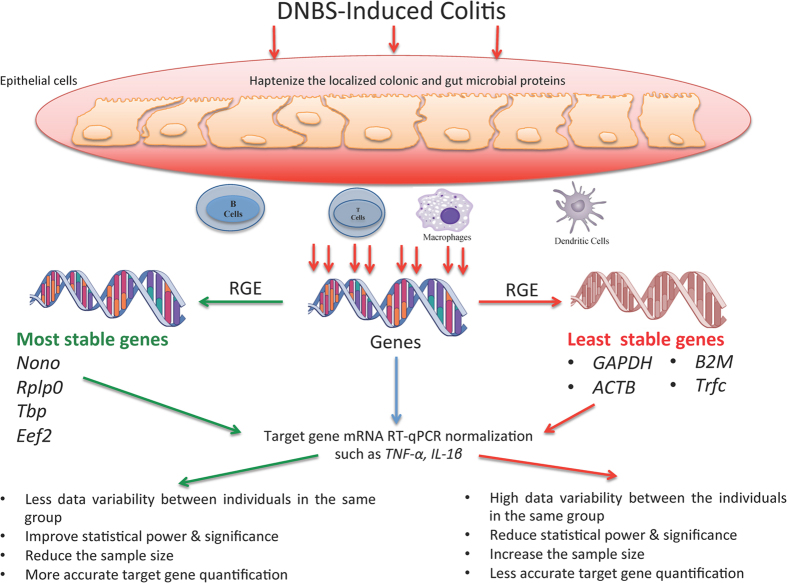
Diagram summary describes the effect of dinitrobenzene sulfonic acid (DNBS) on the reference genes expression (RGE) stability in the gastrointestinal mucosa and their effect when applied to calculate the expression level of target genes using RT-qPCR. Moreover, the immune cellular composition can greatly vary and can contribute to the variation of expression of a given gene.

In conclusion, this study is the first comprehensive analysis referring to the stability of 13 reference genes used in the DNBS mouse model of experimental colitis, and how it could affect the final interpretation and conclusions (Fig. 7). While *Gapdh, B2m*, and *Actb* are the most commonly used reference gene in RT-qPCR experiments, their stability varied widely between the control and DNBS groups that may influence downstream ΔΔCt calculations. In contrast, our comparative study of candidate reference genes suggested the use of *TBP*/*Rplp0* as the appropriate reference genes for target gene normalization in C57BL/6 mice associated with DNBS- experimental colitis model. For all RT-qPCR experiments, we strongly recommend defining a valid reference gene that takes into consideration the specific experimental conditions and the time course that is used, to avoid any misleading results and to support rigorous conclusions.

## Materials and Methods

### Ethics statement

The study was performed at the animal research facility of Basic Medical Sciences Animal Facility (BMSB; University of Manitoba, Winnipeg, Manitoba) and approved by the University of Manitoba Animal Ethics Committee under protocol number 15-010 in accordance with the Guide of Canadian Council on Animal Care in science (CCAC) for the Care and Use of Laboratory Animals for Scientific Purposes.

### Animals & DNBS-Induced Colitis

Male C57BL/6 mice (6–8 weeks old) were purchased from Charles River (Sherbrook, Canada) and maintained in the animal care facility at the University of Manitoba under specific pathogen-free conditions. Mice were anaesthetized using Isoflurane (Abbott, Toronto, Canada). PE-90 tubing (10 cm long; ClayAdam, Parisppany, NJ) that was attached to a tuberculin syringe (BD, Mississauga, Canada) was inserted 3.5 cm into the colon and colitis was induced by intra-rectal administration of 100 μl of 4 mg of DNBS solution (ICN Biomedical Inc. Aurora, OH) in 30% ethanol (Sigma, Mississauga, Canada) and left for 3 days (DNBS + Ethanol 30%, n = 6)^6^,^31^. Controls were time matched and consisted of mice that received intra-rectal administration of 100 μl of 1% phosphate buffer saline (PBS 1%, n = 6) or 100 μl of 4 mg of DNBS solution in 1% PBS (DNBS + PBS 1%, n = 6) or 100 μl 30% Ethanol (Ethanol 30%, n = 6).

### Assessment of colitis severity

To confirm the induction of colitis, the classical inflammatory markers were quantified. The disease activity index (DAI) and weight loss percentage were scored from day 0 to 3 during DNBS treatment, and then mice were sacrificed for macroscopic scoring, as described previously^45^. Colonic myeloperoxidase (MPO) activity level was evaluated using ELISA (Hycult Biotech, PA, USA) and colonic protein levels of IL-1β and TNF-α protein levels were measured using ELISA (R&D Systems, Inc., MN, USA).

### Colonic RNA extraction and cDNA synthesis

Approximately 30–40 mg of colon tissue was used for total RNA extraction using TRIzol^®^ Plus RNA Purification Kit (Life Technologies, NY, USA), according to manufacturer’s instructions. Quality and quantity of RNA were determined by measuring the absorbance at 260 and 280 nm using NanoDrop ND-1000 UV-Vis Spectrophotometer (Thermo Fisher Scientific, Waltham, MA, USA). All samples had an absorption ratio A260/A280 greater than 1.8. RNA (1 μg) from each sample was treated with RQ1 RNase-Free DNase^®^ (Promega Corporation, Madison, WI, USA), according to the manufacturer’s instructions, to remove genomic DNA contamination. Reverse transcription was performed using SuperScript VILO cDNA Synthesis Master Mix (Invitrogen, Grand Island, NY, USA), according to the manufacturer’s instructions, in an Eppendorf Thermo cycler at 25 °C for 10 min, followed by 42 °C for 60 min, and 85 °C for 5 min. Samples were then cooled to 4 °C. cDNA samples were stored at −20 °C for RT-qPCR analysis.

### Primer design

Thirteen candidate reference genes were selected from previously published studies to evaluate their stability in DNBS-induced colitis. The candidate genes were *Gapdh, Actb, β2 m, Hmbs, Hprt, RpLp0, Tbp, Gusb, Ppia, Oaz1, Nono, Tfrc*, and *Eef2*. The primers were designed from nucleotide sequences identified using NCBI BLAST (http://blast.ncbi.nlm.nih.gov/Blast.cgi) to confirm the specificity of the primer design as described previously^15^. The primer characteristics of nominated reference genes are listed in Table 3.

### Quantitative real-time polymerase chain reaction

RT-PCR reactions were performed in a Roch lightCycler 96 Real-Time System using Power SYBR green master mix (Life Technologies, NY, USA), according to the manufacturer’s instructions, in a final volume of 20 μl reactions. To verify the specificity of each primer, a melting-curve analysis was included (65–95 °C with fluorescence measured every 0.5 °C). The absence of contamination from either genomic DNA amplification or primer dimers formation was ensured using two types of controls: the first without reverse transcriptase (no-RT control, one for each RNA) and the second with no DNA template (NTC control, one for each primer pair). All RT-qPCRs were run in duplicate, and the average standard deviation within duplicates of all samples studied was 0.25 cycles.

### Primer’s efficiency

As recommended by the Minimum Information for Publication of Quantitative Real-Time PCR Experiments (MIQE) guidelines^46^, standard curves were generated for each candidate reference gene using the Ct value that resulted from duplicate serial dilutions of cDNA obtained from all experimental groups (n = 12). RT-qPCR efficiencies in the exponential phase were analyzed for each primer pair using standard curves (5-point 5-fold serial dilution of pooled cDNA that included equal amounts from the samples set), the mean Ct values for each serial dilution were plotted against the logarithm of the cDNA dilution factor and calculated according to the equation E = 10^[−1/slope]^ 46, where the slope is the gradient of the linear regression line. The linear dynamic range was determined by the standard curve and correlation coefficients (R^2^) for each gene as reported.

### Reference gene stability assessment

To evaluate the stability of the reference genes in DNBS-induced colitis and corresponding control groups, well-validated assessment logarithms were applied. geNorm defines and ranks the reference genes based on their M value; a lower value of the M average expression stability represents stable expression whereas a high value represents less stable expression^23^. BestKeeper calculated the gene expression stability for candidate genes based on each candidate gene’s Ct values^24^. NormFinder evaluates both intra- and inter-group variations and then combines the two to generate a stability value, which represents a practical measure of the systemic error introduced when investigating the gene^25^. However, comparative delta Ct (ΔCt) compares the relative expression of “pairs of genes” within each sample^26^. Finally, Comprehensive Ranking^27^, which is a web-based comprehensive integrated database, uses a methodology to compare candidate reference gene performance to compensate for weaknesses in the individual tools such as geNorm, Normfinder, BestKeeper and comparative delta Ct method. Comprehensive Ranking, based on the ranking from each tool, gave an appropriate weight to each reference gene and calculated the geometric mean of their weights for the overall final ranking. Raw Ct values (untransformed data) were used directly for data imported from integrated database. These tools take into consideration several parameters, including Ct standard deviation (SD) for respective cDNA detection within different samples.

### Analysis of the appropriateness of selected reference genes in DNBS-experimental colitis using specific targeted genes

Two genes were selected to test the suitability of the 13 reference genes, and to highlight the significance of choosing an optimal reference gene to quantify target gene mRNA expression levels. TNF-α and IL-1β were selected based on their contribution to experimental colitis development. Up-regulation of these cytokines in the inflamed colon has been extensively reported and they are also considered to be classical pro-inflammatory mediators in IBD. Therefore, using the 13 reference genes, the relative *TNF-α* and *IL-1β* mRNA transcript expression in control and DNBS groups was determined by calculating differences in the comparative threshold cycle (ΔΔCt)^13^.

### Selected reference gene stability in DNBS-experimental colitis using External RNA Controls Consortium (ERCC)

External RNA Controls Consortium (ERCC) RNA Spike-In Mixes (Life Technologies, USA) and RNAs from DNBS and control groups were used according to the manufacturer’s instructions. Reverse transcription was performed using SuperScript VILO cDNA Synthesis Master Mix (Invitrogen, Grand Island, NY, USA). To control for template quality, the threshold cycle (Ct) values of 3 selected control ERCC RNAs (ERCC-0002, ERCC-00113, ERCC-0074) were concurrently assessed, and only experimental samples that showed threshold cycles within 2 cycles of the median values of ERCC RNA were engaged in the relative quantification of the reference gene expression levels. Reference gene expression values were calculated by normalizing their expression to the Ct values for ERCC-00113, one of the choices for control ERCC RNAs, using the ΔΔCt method^13^.

### Statistical analysis

The results were compared and analyzed using a one-way/two-way analysis of variance (ANOVA) followed by multiple comparison test. Differences were reported as statistically significant when P < 0.05. GraphPad Prism 6 (GraphPad Software, Inc. La Jolla, CA, USA) was used for statistical procedures and graph plotting.

## Additional Information

**How to cite this article:** Eissa, N. *et al*. Appropriateness of reference genes for normalizing messenger RNA in mouse 2,4-dinitrobenzene sulfonic acid (DNBS)-induced colitis using quantitative real time PCR. *Sci. Rep.* **7**, 42427; doi: 10.1038/srep42427 (2017).

**Publisher's note:** Springer Nature remains neutral with regard to jurisdictional claims in published maps and institutional affiliations.

## Supplementary Material

Supplementary Dataset 1

## Figures and Tables

**Table 1:** Amplification efficiency for selected reference and target genes.

Gene	Slope	R^2^	Efficiency %	Amplification
*Eef2*	−3.55	1.00	99	1.99
*Gapdh*	−3.29	1.00	101	2.01
*Actb*	−3.56	1.00	91	1.91
*B2m*	−3.32	1.00	100	2.00
*TBP*	−3.22	1	104	2.04
*Oaz1*	−3.41	0.99	96	1.69
*Hprt*	−3.47	1	94	1.94
*Nono*	−3.28	0.99	99	1.99
*Ppia*	−3.44	1	95	1.95
*Rplp0*	−3.36	1	98	1.98
*Trfc*	−3.47	1	94	1.94
*Gusb*	−3.53	0.99	92	1.92
*Hmbs*	−3.12	0.99	109	2.09
*TNF-α*	−2.83	0.99	125	2.25
*IL-1β*	−3.30	0.99	101	2.01

**Table 2:** Descriptive performance of candidate reference genes.

Reference Gene	Minimum	Maximum	Median	Mean	Standard Deviation
*Eef2*	17.60	22.73	18.19	18.73	1.405
*Gapdh*	16.38	22.92	18.20	18.48	1.511
*Actb*	14.74	22.63	16.71	16.92	1.634
*B2m*	16.61	23.91	18.81	19.57	1.897
*TBP*	24.72	28.59	25.09	25.22	0.7309
*Oaz1*	31.54	38.14	33.32	33.73	1.808
*Hprt*	20.86	25.78	21.61	21.79	0.9832
*Nono*	21.83	26.27	21.89	22.19	1.431
*Ppia*	16.84	22.87	18.14	18.63	1.761
*Rplp0*	17.58	21.44	18.53	18.56	0.7325
*Trfc*	21.84	29.44	22.99	23.67	2.095
*Gusb*	25.51	32.34	27.46	27.91	1.882
*Hmbs*	23.98	29.88	25.18	25.82	1.637

**Table 3:** Narrative of selected candidate reference and target genes.

Gene Symbol	Gene Name	Gene Function		Sequence 5′->3′	Length	TM	Location	Amplicon Size	Gene Accession Number
GAPDH	Glyceraldehyde3-phosphate dehydrogenase	Glycolysis pathway enzyme	Forward	AGGTCGGTGTGAACGGATTTG	21	62.6	8–28.	95	NM_008084
			Reverse	GGGGTCGTTGATGGCAACA	19	62.6	102–84		
ACTB	Beta Actin	Cytoskeletal structural protein	Forward	GGCTGTATTCCCCTCCATCG	20	61.8	84–103	154	NM_007393
			Reverse	CCAGTTGGTAACAATGCCATGT	22	61.1	237–216		
B2M	Beta 2 Microglobulin	Beta-chain of major histocompatibility complex	Forward	TTCTGGTGCTTGTCTCACTGA	21	61	26–46	104	NM_009735
			Reverse	CAGTATGTTCGGCTTCCCATTC	22	61	129–108		
Hmbs	Hydroxymethylbilane synthase	Heme biosynthetic pathway enzyme	Forward	AAGGGCTTTTCTGAGGCACC	20	62.4	699–718	78	NM_001110251
			Reverse	AGTTGCCCATCTTTCATCACTG	22	60.6	776–755		
Hprt	Hypoxanthine phosphoribosyl transferase	Metabolic salvage of purines	Forward	TCAGTCAACGGGGGACATAAA	21	60.8	324–344	142	NM_013556
			Reverse	GGGGCTGTACTGCTTAACCAG	21	62.4	465–445		
RpLp0	Ribosomal Protein Large P0	Structural constituent of ribosome	Forward	AGATTCGGGATATGCTGTTGGC	22	62.5	290–311	109	NM_007475
			Reverse	TCGGGTCCTAGACCAGTGTTC	21	62.7	398–378		
TBP	TATA-box-binding protein	General transcription factor	Forward	ACCGTGAATCTTGGCTGTAAAC	22	60.8	442–463	86	NM_013684
			Reverse	GCAGCAAATCGCTTGGGATTA	21	61.3	527–507		
Gusb	Glucuronidase, Beta	Lysosomal exoglycosidase	Forward	GGCTGGTGACCTACTGGATTT	21	61.5	722–742	131	NM_010368
			Reverse	GGCACTGGGAACCTGAAGT	19	61.5	852–834		
Ppia	Peptidylprolyl Isomerase A	Protein coding, a cyclosporin binding-protein	Forward	GAGCTGTTTGCAGACAAAGTTC	22	60.2	67–88	125	NM_008907
			Reverse	CCCTGGCACATGAATCCTGG	20	62.6	191–172		
Oaz1	Ornithine Decarboxylase Antizyme 1	Cell growth & Proliferation	Forward	CGCACCATGCCGCTTCTTA	19	63	94–112	169	NM_008753
			Reverse	ATCCCGCTGACTGTTCCCT	19	62.6	262–244		
Nono	Non-POU Domain Containing, Octamer-Binding	Transcriptional regulation and RNA splicing	Forward	AAAGCAGGCGAAGTTTTCATTC	22	60	301–322	77	NM_023144
			Reverse	ATTTCCGCTAGGGTTCGTGTT	21	61.7	377–357		
Tfrc	Transferrin Receptor	Endocytosis & HIF1 signalling pathway	Forward	GTTTCTGCCAGCCCCTTATTAT	22	60.1	1495–1516	152	NM_011638
			Reverse	GCAAGGAAAGGATATGCAGCA	21	60.7	1646–1626		
Eef2	Eukaryotic Translation Elongation Factor 2	Protein Synthesis	Forward	TGTCAGTCATCGCCCATGTG	20	62.2	65–84	123	NM_007907.2
			Reverse	CATCCTTGCGAGTGTCAGTGA	21	62.1	187–167		
TNF-α	Tumor Necrosis Factor Alpha	Pro-inflammatory cell signalling protein (Cytokine)	Forward	CCCTCACACTCAGATCATCTTCT	23	60.9	230–252	61	NM_013693
			Reverse	GCTACGACGTGGGCTACAG	19	62.1	290–272		
IL-1β	Interleukin (IL)-1 β	Pro-inflammatory cell signalling protein	Forward	GCAACTGTTCCTGAACTCAACT	22	60.7	4–25	89	NM_008361
			Reverse	ATCTTTTGGGGTCCGTCAACT	21	61.4	92–72		
