# Supplementary material for: Appropriateness of reference genes for normalizing messenger RNA in mouse 2,4-dinitrobenzene sulfonic acid (DNBS)-induced colitis using quantitative real time PCR
Source: Sci Rep. 2017 Feb 10;7:42427. doi: 10.1038/srep42427 (PMC5301225; doi:10.1038/srep42427)
Supplement: Supplementary Dataset 1 [file srep42427-s1.pdf]

Appropriateness of reference genes for normalizing messenger RNA in mouse 2,4-dinitrobenzene sulfonic acid (DNBS)-induced colitis using quantitative real time PCR

Nour Eissa[1,2*], Laëtitia Kermarrec[1*], Hayam Hussein[3], Charles N. Bernstein[4,5], Jean-Eric Ghia[1,2,4,5]

[1]Immunology, University of Manitoba, Winnipeg, MB, Canada

[2]Children's Hospital Research Institute of Manitoba, University of Manitoba, Winnipeg, MB, Canada

[3]Large Animal Medicine, William R. Pritchard Veterinary Medical Teaching Hospital, School of Veterinary Medicine, University of California Davis, CA, USA

[4]Internal Medicine section of Gastroenterology, University of Manitoba, Winnipeg, MB, Canada

[5]IBD Clinical and Research Centre, University of Manitoba, Winnipeg, MB, Canada

* First co-authorship, authors contributed equally

Corresponding address:

Dr. Jean-Eric Ghia, Department of Immunology, College of Medicine, University of Manitoba, 431 Apotex Centre, 750 McDermot Avenue, Winnipeg, MB R3E 0T5 Canada Tel: +1 (204) 789-3802, Fax: +1 (204) 789-3921 Email: Jean-Eric.Ghia@umanitoba.ca or jeghia@yahoo.fr

Supplementary figures legend

Figure S1. Amplification efficiency of primers reference gene candidates. The X-axis denotes the log10 cDNA dilution series, and the Y-axis denotes the cycle threshold (Ct). The primer efficiency (E) is computed by [10(1/-S)-1] × 100%, where S represents the slope of the linear regression line. ROCH Light Cycler Software was used for analysis.

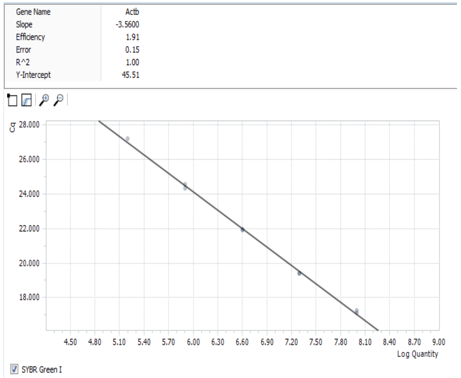
Gene Name Actb
Slope -3.5600
Efficiency 1.91
Error 0.15
R^2 1.00
Y-Intercept 45.51
Log Quantity
SYBR Green I

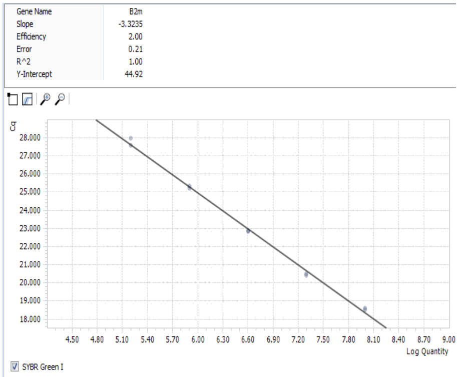
Gene Name B2m
Slope -3.3235
Efficiency 2.00
Error 0.21
R^2 1.00
Y-Intercept 44.92
Log Quantity
SYBR Green I

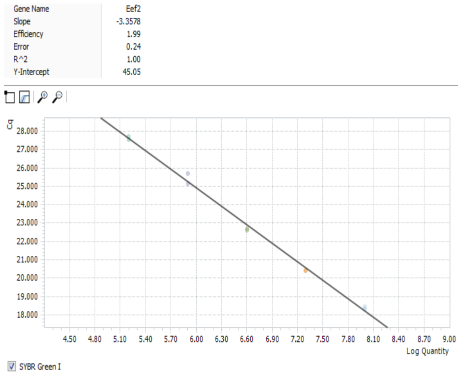
Gene Name Eef2
Slope -3.3578
Efficiency 1.99
Error 0.24
R^2 1.00
Y-Intercept 45.05
Log Quantity
SYBR Green I

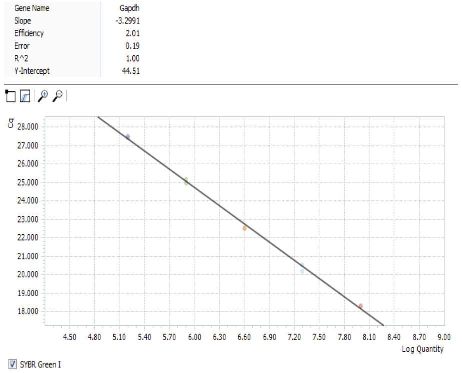
Gene Name Gapdh
Slope -3.2991
Efficiency 2.01
Error 0.19
R^2 1.00
Y-Intercept 44.51
Log Quantity
SYBR Green I

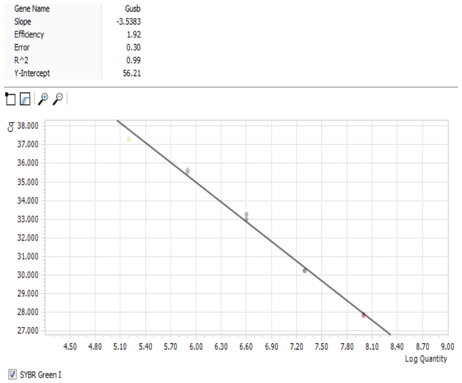
Gene Name Gusb
Slope -3.5383
Efficiency 1.92
Error 0.30
R^2 0.99
Y-Intercept 56.21
Log Quantity
SYBR Green I

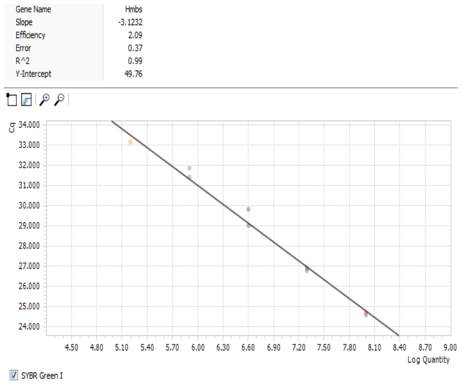
Gene Name Hmbs
Slope -3.1232
Efficiency 2.09
Error 0.37
R^2 0.99
Y-Intercept 49.76
Log Quantity
SYBR Green I

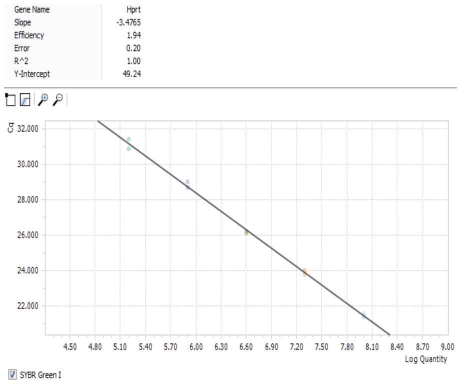
Gene Name Hprt
Slope -3.4765
Efficiency 1.94
Error 0.20
R^2 1.00
Y-Intercept 49.24
Log Quantity
SYBR Green I

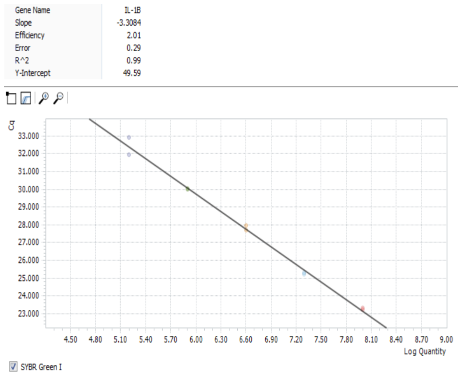
Gene Name IL-1B
Slope -3.3084
Efficiency 2.01
Error 0.29
R^2 0.99
Y-Intercept 49.59
Log Quantity
SYBR Green I

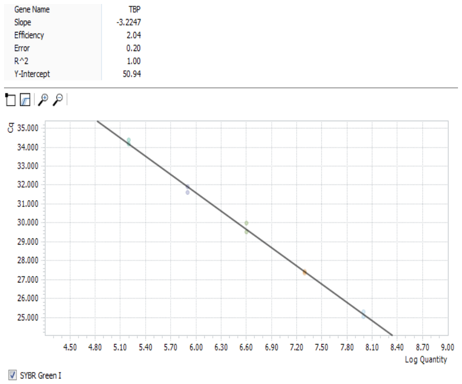
Gene Name TBP
Slope -3.2247
Efficiency 2.04
Error 0.20
R^2 1.00
Y-Intercept 50.94
Log Quantity
SYBR Green I

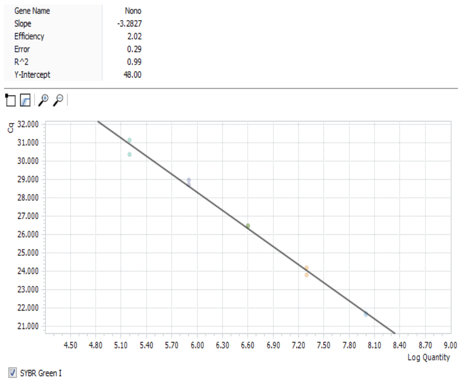
Gene Name Nono
Slope -3.2827
Efficiency 2.02
Error 0.29
R^2 0.99
Y-Intercept 48.00
Log Quantity
SYBR Green I

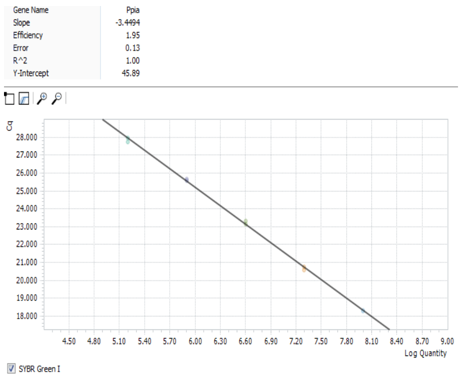
Gene Name Ppia
Slope -3.4494
Efficiency 1.95
Error 0.13
R^2 1.00
Y-Intercept 45.89
Log Quantity
SYBR Green I

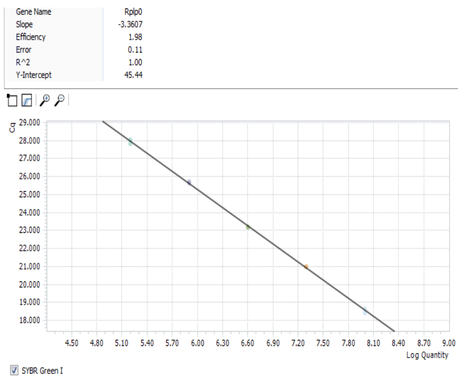
Gene Name Rplp0
Slope -3.3607
Efficiency 1.98
Error 0.11
R^2 1.00
Y-Intercept 45.44
Log Quantity
SYBR Green I

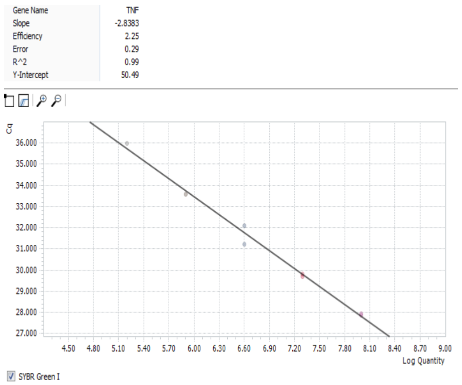
Gene Name TNF
Slope -2.8383
Efficiency 2.25
Error 0.29
R^2 0.99
Y-Intercept 50.49
Log Quantity
SYBR Green I

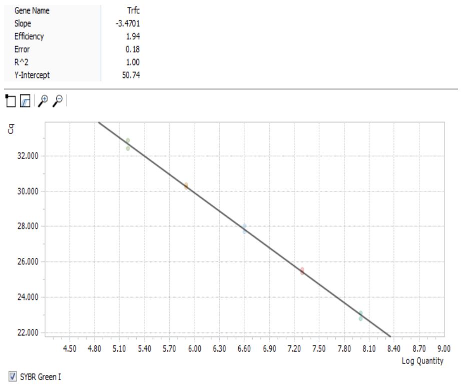
Gene Name Trfc
Slope -3.4701
Efficiency 1.94
Error 0.18
R^2 1.00
Y-Intercept 50.74
Log Quantity
SYBR Green I

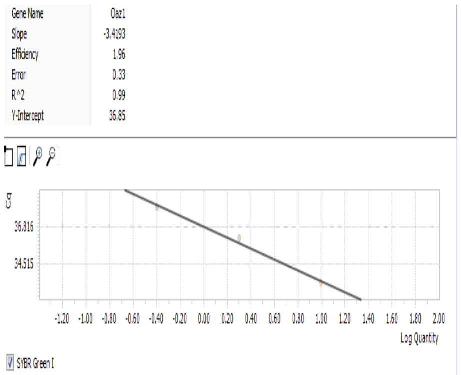
Gene Name Oaz1
Slope -3.4193
Efficiency 1.96
Error 0.33
R^2 0.99
Y-Intercept 36.85
Log Quantity
SYBR Green I
